# Thiazide diuretics alone or in combination with a potassium-sparing diuretic on blood pressure-lowering in patients with primary hypertension: protocol for a systematic review and network meta-analysis

**DOI:** 10.1186/s13643-022-01890-y

**Published:** 2022-02-08

**Authors:** Vítor M. Martins, Patrícia K. Ziegelmann, Lucas Helal, Filipe Ferrari, Marcelo B. Lucca, Sandra C. Fuchs, Flávio D. Fuchs

**Affiliations:** 1 Graduate Program in Cardiology and Cardiovascular Sciences, School of Medicine, Universidade Federal do Rio Grande do Sul, Porto Alegre, RS Brazil; 2 Division of Cardiology, Hospital de Clínicas de Porto Alegre, R. Ramiro Barcellos 2350, Porto Alegre, RS 90035–903 Brazil; 3 Graduate Program in Epidemiology, School of Medicine, Universidade Federal do Rio Grande do Sul, Porto Alegre, RS Brazil; 4 Center for Journalology, Ottawa Hospital Research Institute, Ottawa, Canada; 5 INCT PREVER, Clinical Research Center, Hospital de Clinicas de Porto Alegre, Porto Alegre, RS Brazil

**Keywords:** Hypertension, Drug therapy, Diuretic, Thiazide, Potassium-sparing

## Abstract

**Background:**

The use of thiazide (T) diuretics for the treatment of hypertension may be associated with adverse metabolic effects, which can be minimized by combining thiazides with potassium-sparing (PS) diuretics. The additional blood pressure (BP)-lowering effect provided by the addition of a PS diuretic is unclear. Due to a large number of drugs in the T diuretics class, and the possible difference between them, there is a need to identify the best available evidence for health decision-making. This systematic review with network meta-analysis aims to compare the antihypertensive efficacy of T diuretics alone or in combination with a PS diuretic in patients with primary hypertension, as well as the safety of such drugs through the measurement of drug-related adverse events.

**Methods:**

A comprehensive electronic search will be conducted in six electronic bibliographic databases (PubMed/MEDLINE, Cochrane Library, Embase, Web of Science, Scopus, Lilacs), a registration database (ClinicalTrials.gov), and Educational Resources Information Center (ERIC [ProQuest]), published from inception to the date of the search. The search will be updated towards the end of the review. A hand search of the reference sections of the included studies and cited studies will also be performed. In case of missing data, authors will be contacted by e-mail or academic social networking sites whenever possible. To be included in the review, studies must be double-blind randomized controlled trials evaluating T diuretics alone or in combination with PS diuretics in patients with primary hypertension. The primary outcome measure will be office BP. Ambulatory BP monitoring (ABPM), non-melanoma skin cancer, major adverse cardiovascular events, laboratory parameters, and the number of withdrawals will be included as secondary outcomes. The results will be quantitatively summarized using differences between the mean change from baseline or differences between means for quantitative outcomes and relative risk for dichotomous outcomes. Results will be presented as mean or relative risk with credible intervals through a league table. The treatments will also be ranked using the surface under the cumulative ranking curve method. The risk of bias will be assessed through the RoB 1.0 tool.

**Discussion:**

To the best of our knowledge, this review will be the first to synthesize currently available evidence on the antihypertensive efficacy of different T diuretics alone or in combination with PS diuretics in adults with hypertension. The goals of hypertension treatment are to control high BP and to reduce associated cardiovascular morbidity and mortality, using the most appropriate therapy. Thiazides are widely used for pharmacological treatment due to their demonstrated effectiveness in reducing BP, favorable safety profile, and low cost. The results of this study will provide evidence regarding the best therapeutic strategies with T and PS diuretics, evidencing interventions with better antihypertensive efficacy and safety profile.

**Trial registration:**

This systematic review and network meta-analysis was prospectively registered at the PROSPERO database (CRD42018118492).

**Supplementary Information:**

The online version contains supplementary material available at 10.1186/s13643-022-01890-y.

## Background

Thiazide (T) diuretics have been used for the treatment of hypertension for more than five decades, being among the first oral antihypertensive agents with an acceptable side-effect profile [1, 2]. Agents of this class derived from benzothiadiazine are called “thiazide-type diuretics,” such as hydrochlorothiazide and bendroflumethiazide. Drugs with a similar pharmacologic action on the kidney but that do not have the thiazide chemical structure (e.g., indapamide, chlorthalidone, and metolazone) are termed “thiazide-like diuretics” and are recognized together with T diuretics as a class of blood pressure (BP)-lowering agents.

T diuretics have been demonstrated to be effective at low doses [3–10], where the steepest part of the dose-response curve is typically seen in patients with primary hypertension [11]. Chlorthalidone and indapamide have provided greater antihypertensive efficacy than hydrochlorothiazide, a thiazide-type diuretic, at similar dose levels [12–16]. Chlorthalidone is 1.5 to 2 times more effective than hydrochlorothiazide in reducing BP at the same dose [13]. The lower efficacy of hydrochlorothiazide may be explained by a shorter duration of action compared to chlorthalidone and indapamide [13, 14, 17].

The use of T diuretics may be associated with adverse metabolic effects, especially hypokalemia and hyperglycemia, but also hyponatremia, hyperuricemia, hyperlipidemia, and hypomagnesemia [3, 18, 19]. The incidence of these metabolic effects occurs in a dose-response manner [3, 11, 20]. The use of high doses of thiazide-type diuretics without a potassium-sparing (PS) diuretic was identified as a risk factor for sudden death [21]. The risk of hypokalemia may be minimized by combining thiazides with PS diuretics—mineralocorticoid receptor antagonists (e.g., spironolactone and eplerenone) or blockers of the epithelial sodium channel (e.g., amiloride and triamterene), which may also mitigate the potential for impaired glucose tolerance associated with thiazides [22]. Nonetheless, PS diuretics may also have some side effects, such as hyperkalemia, and spironolactone have been associated with gynecomastia [23].

Although the antihypertensive properties of spironolactone and eplerenone have been well documented [24–28], the BP-lowering effect of amiloride and triamterene has not been as clearly determined. A previous systematic review reported no significant effects on BP at low doses of amiloride and triamterene [29]. In contrast, some studies suggest that amiloride may be effective in resistant hypertension [30] and may have a stronger antihypertensive effect at higher doses in non-resistant hypertension [22, 31].

It remains to be clarified whether different clinical outcomes are dependent on diuretic presentation. Both chlorthalidone and indapamide have been shown to reduce cardiovascular events in benchmark randomized trials [32, 33], whereas there is no evidence that hydrochlorothiazide alone reduces cardiovascular events [34]. There are no randomized controlled trials (RCTs) that directly compared different T diuretics (alone or in combination with PS diuretics) on cardiovascular outcomes in patients with hypertension, and previous indirect comparisons by meta-analyses and evidence from observational studies provided conflicting results [35–39]. Given the plethora of presentation types among thiazides, no between-drugs comparison has been conducted at the level of a primary study—RCT, whereas decision-makers may need the best evidence to choose the first-line therapy when opting by thiazides. Since substantial clinical evidence concluded that the intensity of BP reduction is the major determinant of reduction in cardiovascular risk in hypertensive patients [11, 40–42], the BP-lowering effect among diuretics becomes an appropriate surrogate outcome. Although the adverse effects of T diuretics can be minimized with the addition of a PS diuretic, little is known about the additional BP-lowering effect of this association. For this purpose, a network meta-analysis (NMA) of RCTs seems to be justifiable. The use of NMA models will allow comparisons of all available drugs even if they were not included in the same RCT. Moreover, treatments will be ranked according to each outcome of interest.

The systematic review with NMA will be conducted through a Bayesian mixed treatment comparison model to compare the efficacy of T diuretics alone or in combination with a PS diuretic in patients with primary hypertension, as well as the safety of such drugs through the measurement of drug-related adverse events.

### Objectives

The purpose of this systematic review and NMA is to investigate, summarize, and compare quantitatively the following: (a) the BP-lowering efficacy of T diuretics alone or in combination with PS diuretics in patients with primary hypertension (*primary objective*) and (b) the impact of the T diuretics alone or in combination with a PS diuretic about laboratory parameters, non-melanoma skin cancer, major adverse cardiovascular events (MACE), and withdrawals (due to adverse effects and for any reason) (*secondary objectives*). Both primary and secondary objectives are listed in the “Outcomes and prioritization” section.

## Methods

The protocol of this systematic review with NMA was written guided by PRISMA-P statement [43] (see checklist in Additional file 1), and the PRISMA Explanation and Elaboration article for guidance [44]. The first draft of this protocol has been prospectively registered in the International Prospective Register of Systematic Reviews—PROSPERO (CRD42018118492) [45].

### Eligibility criteria

#### Participants

Adults (18 years or older), regardless of sex and race, are diagnosed with primary hypertension (as stated by the authors). To minimize a possible carryover effect, only studies in which participants were at least 2 weeks without active antihypertensive treatment before randomization will be included.

#### Interventions

Antihypertensive agents from the class of diuretics are as follows:

- Thiazide diuretics alone, specifically: hydrochlorothiazide, chlorothiazide, butizide, bendroflumethiazide, hydroflumethiazide, trichlormethiazide, methyclothiazide, polythiazide, cyclothiazide, cyclopenthiazide, chlorthalidone, metolazone, quinethazone, fenquizone, clorexolone, clopamide, indapamide, diapamide, isodapamide, mefruside, xipamide, bemetizide, benzthiazide, and chlorazanil
- Thiazide diuretics in combination with a potassium-sparing diuretic, specifically: spironolactone, eplerenone, amiloride, and triamterene.

T diuretics alone and T diuretics in combination with PS agents will be called “Treatment T” and “Treatment TPS,” respectively. Additionally, the treatments will be classified according to the mean daily dose. The doses of each T diuretic will be categorized as proportions of the manufacturer’s recommended starting dose. In the case where a range of starting doses is recommended by the manufacturer, the lowest dose will be considered to be the starting dose (1×). Therefore, both (Treatment T and Treatment TPS) will be categorized into two groups, according to the mean daily dose of the thiazide component: low dose (< 2× start dose) and high dose (≥ 2× start dose). An exception to this rule will be applied to hydrochlorothiazide. Although hydrochlorothiazide has the same recommended starting dose as chlorthalidone (12.5 mg/day), the available literature suggests that chlorthalidone is 1.5 to 2 times as effective as hydrochlorothiazide in lowering BP at the same dose [13]. For this reason, the initial dose of hydrochlorothiazide will be considered as 25 mg/day.

#### Comparators

The eligible interventions will be compared among themselves. Besides, to expand the geometry of the network, treatments out of interest, but connected with the ones of interest adding indirect comparisons for the network, will be included as common comparators. The ones considered are placebo or any other antihypertensive drug, alone or in combination, regardless of the pharmacological class. As potassium chloride may have an antihypertensive effect, thiazides with potassium supplementation will not be considered an eligible intervention, but this combination may also be eligible as a comparator. Treatments to be included as common comparators will also be grouped into categories based on their pharmacological class and mean daily dose.

#### Outcomes

Studies will have to measure at least one of the primary outcomes or one of the secondary outcomes.

#### Primary outcomes

Office systolic and diastolic BP.

#### Secondary outcomes

- Efficacy outcomes

ABPM will be quantitatively synthesized considering data from daytime, nighttime, and 24-h BP (systolic and diastolic).

MACE will be quantitatively synthesized as a composite outcome and also individually whenever reported.

- Safety (harms) outcomes

Laboratory parameters will be analyzed quantitatively.

Non-melanoma skin cancer will be analyzed qualitatively.

The number of withdrawals (due to adverse effects and for any reason) among the eligible treatments will be analyzed qualitatively.

#### Study designs

Only double-blind randomized controlled (placebo or other active treatment) trials will be included as the unit of analysis. Studies will be limited for those beginning with 3 weeks of follow-up last to 52 weeks since trials designed with longer follow-up often target primordial cardiovascular outcomes (e.g., cardiovascular mortality).

Studies with step-up therapy in non-responders (i.e., the addition of another antihypertensive drug as second-line therapy in patients not meeting a target goal BP level) will be included, as long as pre-step-up BP measurements are provided.

Crossover studies will be included entirely if there is a clear report of at least 2 weeks of washout among the treatments tested. If not, only the first period of the study will be included, as long as pre-crossover data are provided. Factorial designs will be also considered whenever interaction between treatments is absent.

No restriction will be imposed for the language or date of publication, publication status, or sample size. Whenever possible, any report of RCTs (e.g., conference abstracts) in which partial data are sufficient to be analyzed (quantitatively or qualitatively) will be included.

### Exclusion criteria

Trials with patients with the following conditions will be excluded: heart failure with reduced and preserved ejection fraction (≤ 40% and ≥ 50%, respectively) and New York Heart Association functional class II–IV; chronic renal disease requiring dialysis; or a documented serum creatinine level more than 1.5 times the normal range, due to the lower effectiveness of T diuretics in patients with impaired kidney function [46]. Drugs with BP-lowering effect indicated for other diseases (e.g., doxazosin for benign prostatic hyperplasia) will be excluded.

### Information sources

#### Electronic searches

For an extensive and comprehensive survey of the literature, the search strategy will be performed in six electronic bibliographic databases (PubMed/MEDLINE, Cochrane Library, Embase, Web of Science, Scopus, Lilacs), a registration database (ClinicalTrials.gov) for potential results in unpublished studies and Educational Resources Information Center (ERIC [ProQuest]) for results in non-indexed journals or other forms of reporting (thesis, conference summary, monograph, etc.), from database inception to the date of the search. The search will be updated towards the end of the review. Hand searches will also be performed of the reference lists of retrieved papers and previous systematic reviews. For articles not published in English, Spanish, or Portuguese, Google Translator will be used.

#### Search strategy

The main electronic search strategy was designed for MEDLINE and will be adapted as appropriate for each of the databases. Literature search strategies will be developed using MeSH terms and their synonyms, and boolean operators (where possible) to improve searches. Keywords and terms of MeSH include the following: “hydrochlorothiazide,” “chlorothiazide,” “bendroflumethiazide,” “hydroflumethiazide,” “trichlormethiazide,” “methyclothiazide,” “polythiazide,” “cyclopenthiazide,” “chlorthalidone,” “metolazone,” “clopamide,” “indapamide,” “mefruside,” “xipamide,” “bemetizide,” “benzthiazide,” “chlorazanil,” “spironolactone,” “eplerenone,” “amiloride,” “triamterene,” “thiazide diuretics,” “inhibitor of the epithelial sodium channel,” “potassium sparing diuretic,” and “hypertension.” Comprehensive search strategies for all the bases that will be consulted are included in Additional file 2.

### Study records

#### Data management

After the queries, each electronic database will be exported to a reference manager software (EndNote X9) and duplicates will be removed. Other found sources will be inserted manually in the reference manager and checked again for duplicates. Then, titles and abstracts will be stored at the reference manager till the beginning of the eligibility process. Potentially eligible titles and abstracts and the excluded ones will be stored in specific folders. The physical report will be scanned for future purposes or independent researchers checking and deposited in the Google Drive® with specific folders for inclusion and exclusion reasons. A final list of included and excluded articles in each step will be recorded. If a trial has unpublished outcomes of BP, authors will be contacted to seek any potential information. Then, the data will be extracted and stored in a piloted spreadsheet for data synthesis.

#### Selection process

The screening for eligible RCTs will be conducted in a two-step manner. First, the reports will be checked on the level of titles and abstracts. For this purpose, the liberal accelerated approach will be carried out [47], in which one author will flag the potentially eligible reports and the excluded ones, and a second author will review records excluded by the first reviewer. Disagreements will be solved by consensus. On the level of the titles and abstracts, the reports will be stored in two folders: one for potentially eligible reports and another for the excluded ones.

**Fig. 1:**
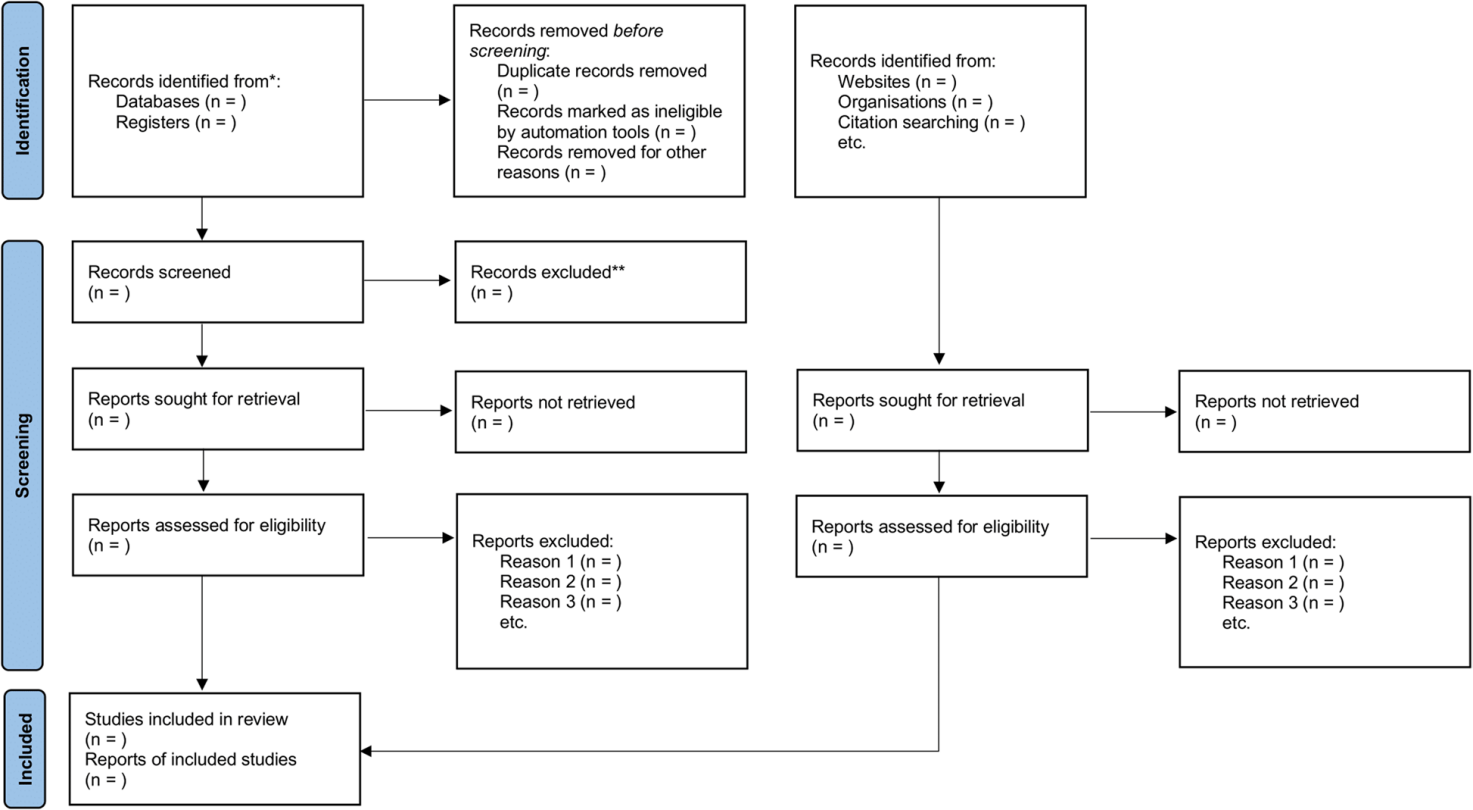
PRISMA flow diagram

The remaining potential eligibility records will be checked by their full texts in duplicate by pairs of independent reviewers. Disagreements will be solved by consensus or by a third reviewer’s decision. Reports will be flagged as eligible or ineligible with their respective reasons. In case of any physical report to be checked, they will be separated in the same manner as digital records after the final decision, but they will be checked for eligibility directly by the full-text assessment. The results of the selection process will be presented in a flow diagram, as shown in Fig. 1.

#### Data collection process

Data extraction will be done in duplicate, with independent reviewers through a piloted data extraction form. The piloting of the form will be done by two experienced reviewers with the first 3 eligible records and amendments will be made accordingly to the process. Disagreements will be solved by the opinion of a third reviewer. Reasons for amendments and versions of the data extraction form will be recorded.

### Data items

For quantitative outcomes, the target data to be extracted will be the mean change from baseline with standard deviation or standard error or confidence interval or *p*-value and sample size. Also, the mean and standard deviation will be extracted at baseline and follow-up to calculate change from baseline standard deviation considering zero correlation. When none of those strategies were available, change from baseline standard deviation will be imputed using the mean value from the one available for the same treatment group. For dichotomous outcomes, the number of events and the sample size randomized for each treatment arm will be collected. In case of missing information and before imputation, authors will be contacted by e-mail or academic social networking sites (e.g., ResearchGate) whenever possible.

#### Study characteristics

First author, year of publication, acronym, publication type, study design (parallel, crossover, factorial), washout period (weeks), study period (weeks), number of patients randomized (*n*), industry sponsorship, country, the language of publication, BP measurement (peak, trough), BP position (sitting, standing, supine).

#### Patient baseline characteristics

Age (years), gender (male/female, %), race (white, black, other), body mass index—BMI (kg/m^2^).

#### Interventions and comparators

Name of the thiazide, an initial daily dose of thiazide, mean daily dose of thiazide at the end of the study, name of the thiazide association (with PS diuretic), an initial daily dose of the thiazide association, mean daily dose of the thiazide association, name of the comparator, initial daily dose of the comparator, and mean daily dose of the comparator.

### Outcomes and prioritization

#### Primary outcomes

- Office systolic and diastolic BP: measured as continuous outcomes at baseline and follow-up. For both, baseline and follow-up time, if BP measurements are available at more than a single time-point during the 24 h, only the trough measurement will be used, if available. Trough BP is defined as the BP measurement taken before the next dosing schedule. If the timing of measurement is not reported, BP will be assumed to have been taken at the trough. When BP measurement data are available in more than one position, sitting BP will be the first preference, followed by standing and supine position. If BP measurements are available more than once within the accepted follow-up window, the last measurement will be used. If patients in the study receive a force-titrated dose, the BP measurements under the highest administered dose will be included. Studies in which BP measurements were not taken under resting conditions will be excluded. Also, the mean and standard deviation will be extracted at baseline and follow-up. Missing standard deviations will be imputed using the mean value from the ones available for the same treatment group. When the mean change from baseline was not available it will be computed using data from baseline and follow-up using zero for the correlation between before and after values (this is a conservative approach).

#### Secondary outcomes

- Laboratory parameters (serum potassium, serum total cholesterol, serum HDL-C, serum LDL-C, serum triglycerides, fasting plasma glucose, and HbA1c). The data to be extracted also will be the mean change from baseline as for the primary outcomes. Serum potassium will be presented and synthesized in mEq/L. Fasting plasma glucose, lipid profile, and uric acid will be presented and synthesized in mg/dL. HbA1c will be presented in percentage. Whenever necessary, transformations will be carried on
- Ambulatory systolic and diastolic BP: mean at daytime, mean at nighttime, and mean during the 24 h. Data to be extracted are the mean values, standard deviation, and sample size. Missing standard deviations will be imputed as described before.
- Non-melanoma skin cancer: The data to be extracted are the sample size randomized and the number of events.
- MACE: all-cause mortality, cardiovascular mortality, fatal stroke, non-fatal stroke, fatal myocardial infarction, non-fatal myocardial infarction, and hospitalization due to heart failure. The data to be extracted are the sample size randomized and the number of events.
- Withdrawals: the number of patients who withdrew due to adverse effects and for any reason will be recorded.

### Risk of bias in individual studies

For the assessment of the risk of bias of included studies, the Cochrane Collaboration spreadsheet settled for the Risk of Bias 1.0 tool [48] will be used through the Review Manager software (RevMan version 5.3), and final decisions will be stored at the RoB 1.0 spreadsheet. All of the materials used in this systematic review with NMA will be shared thereafter in a public repository, after the publication of the manuscript.

### Data synthesis

#### Main analyses

The results will be quantitatively summarized using differences between mean change from baseline or differences between means for quantitative outcomes and relative risk for dichotomous outcomes. Traditional meta-analysis will be carried out for each pair of comparisons. Since heterogeneity is expected, random effects models will be used. Heterogeneity will be measured using the *I*^2^ statistics. To estimate between-study heterogeneity, the DerSimonian & Laird estimator will be used. For continuous outcomes, the pooled effect size will be estimated using the inverse of variance method and to the dichotomous outcomes, the Mantel-Haenszel method. To minimize heterogeneity, treatments were grouped based on dose.

To compare all the thiazides classes (Treatment T low dose, Treatment T high dose, Treatment TPS low dose, Treatment TPS high dose) and placebo quantitatively, a multiple treatment comparison (MTC) NMA models will be run for each outcome combining all available direct and indirect evidence from pairs of treatments. This will be made through the generalized Bayesian linear model proposed by Lu and Ades [49]. For this, prior will be non-informative and the nature of the effect sizes will be considered to choose the likelihood. Effect sizes will then be estimated by Markov chain Monte Carlo simulation. A number of simulations and thin values will be chosen by checking autocorrelation plots and trace plots. Both, fixed and random effect models with homogeneity of variance will be adjusted and the deviance information criterion (DIC) value will be used to decide between them. Analysis of inconsistency, when necessary, will be performed using the split node method. Results will be presented as mean differences or relative risk with credible intervals through a league table. Also, a frame with the geometry of comparisons will be provided. The classes of treatment will also be ranked using the surface under the cumulative ranking (SUCRA) curve method. A narrative description will be provided with the information presented in the text and tables to summarize the characteristics and findings of the included studies.

All the statistical analyses will be carried on using the R software (v. 3.5.2) using the packages “meta,” “metafor,” and “gemtc” (that nest the WinBUGS software to the R Package).

#### Pre-planned sensitivity analyses

Analyses in separated (industry-sponsored vs non-industry sponsored) are planned to explore the sensitivity of the results.

Note: being an exploratory analysis by nature, other sensitivity analyses can be carried out and will be displayed as deviations from the protocol in the final report. Disclaimer: no conclusion or recommendation will be done based on exploratory analyses.

### Checking for asymmetry and suggestion of publication bias

The asymmetry of results will be investigated for each pair of interventions (as long as they have ten or more studies) through a contour plot in which point estimates will be inserted against the inverse of their standard error (e.g., a funnel plot). The Begg’s and Egger’s tests will provide statistical support to any judgment and assessment.

### Confidence in cumulative evidence

Assessment of the quality of evidence is not planned as part of this study.

## Discussion

Hypertension is a major worldwide public health problem, being the main cause of cardiovascular disease. T diuretics are commonly recommended as first-line treatment for hypertension due to good tolerability and proven BP-lowering efficacy. Their use may be associated with adverse metabolic effects, which may be minimized by combining thiazides with PS diuretics. It remains unknown whether different diuretics are associated with different clinical outcomes. This systematic review with NMA will compare the antihypertensive efficacy of T diuretics alone or in combination with a PS diuretic in patients with primary hypertension, as well as the safety of such drugs through the measurement of drug-related adverse events. The protocol of this NMA was written guided by the PRISMA-P statement, which will endorse the transparency, accuracy, and completeness of the study. Multiple databases will be searched to ensure a comprehensive review, and no restriction will be imposed for the language or date of publication, publication status, or sample size. This study is in accordance with the compliance of the reproducibility standards, as the authors intend to publish the results in an open-access journal, and all materials, search strategies, raw and treated data, statistical code, and outputs will be publicly shared.

As there are no RCTs directly comparing the efficacy and safety of different T alone and in combination with PS diuretics, the results of this NMA will provide evidence-based information to improve policymaking for patients with hypertension.

## Supplementary Information

**Additional file 1.** PRISMA-P 2015 Checklist.

**Additional file 2.** Search strategies.

## Data Availability

The authors intend to publish the results in an open-access journal (but not limited to), indexed in the Directory of Open Access Journals, with the copyrights transferred to the authors. Also, all materials, search strategies, raw and treated data, statistical code, and outputs will be publicly shared without restrictions to access the data or expiration date. The repository was not chosen yet and will be provided in further amendments or the final report of this study.

## Abbreviations

PRISMA-P: Preferred Reporting Items for Systematic Reviews and Meta-Analyses Protocols
CNPq: National Council for Scientific and Technological Development
CAPES: Coordination for the Improvement of Higher Education Personnel
BP: Blood pressure
NMA: Network meta-analysis
HbA1c: Glycated hemoglobin
LDL-C: Low-density lipoprotein cholesterol
HDL-C: High-density lipoprotein cholesterol
MACE: Major adverse cardiovascular events
ACE: Angiotensin-converting enzyme
ARBs: Angiotensin receptor blockers
BBs: Beta-blockers
CCBs: Calcium channel blockers
ABPM: Ambulatory blood pressure monitoring
BMI: Body mass index
MTC: Multiple treatment comparison
DIC: Deviance information criterion
SUCRA: Surface under the cumulative ranking
