# Supplementary material for: Thiazide diuretics alone or in combination with a potassium-sparing diuretic on blood pressure-lowering in patients with primary hypertension: protocol for a systematic review and network meta-analysis
Source: Syst Rev. 2022 Feb 8;11:23. doi: 10.1186/s13643-022-01890-y (PMC8826711; doi:10.1186/s13643-022-01890-y)
Supplement: Supplementary file 2 — Additional file 2. Search strategies. [file 13643_2022_1890_MOESM2_ESM.docx]

**Additional file 2**

**Search strategies**

**PUBMED**

#1 hydrochlorothiazide[MeSH] OR chlorothiazide[MeSH] OR dichlothiazide OR dihydrochlorothiazide OR hctz OR butizide OR buthiazide OR isobutylhydrochlorothiazide OR bendroflumethiazide[MeSH] OR bendrofluazide OR hydroflumethiazide[MeSH] OR trifluoromethylhydrothiazide OR trichlormethiazide[MeSH] OR methyclothiazide[MeSH] OR polythiazide[MeSH] OR cyclothiazide OR cyclopenthiazide[MeSH] OR cyclomethiazide OR chlorthalidone[MeSH] OR chlortalidone OR chlorphthalidolone OR metolazone[MeSH] OR phthalamudine OR quinethazone OR metolazone OR quinethazone OR fenquizone OR clorexolone OR chlorexolone OR clopamide[MeSH] OR indapamide[MeSH] OR metindamide OR diapamide OR mefruside[MeSH] OR xipamide[MeSH] OR bemetizide OR benzthiazide OR benzothiazide OR chlorazanil OR thiazide OR diuretics, thiazide[MeSH] OR thiazide diuretics[MeSH] OR benzothiadiazine diuretic[MeSH] OR sodium chloride symporter inhibitors OR sodium chloride cotransporter inhibitor OR potassium depleting diuretics OR diuretics, potassium depletion OR triamterene[MeSH] OR amiloride[MeSH] OR spironolactone[MeSH] OR eplerenone OR sodium channel blockers OR EnaC blocker OR inhibitor of the epithelial sodium channel[MeSH] OR co-amilozide OR coamilozide OR aldosterone receptor antagonist[MeSH] OR aldosterone antagonist OR mineralocorticoid antagonist OR mineralocorticoid receptor antagonist OR potassium sparing diuretic[MeSH]

#2 hypertension[MeSH] OR “hypertensive patients”[tw] OR “patients, hypertensive” OR “blood pressure”[tiab] OR “systolic blood pressure”[tiab] OR “diastolic blood pressure”[tiab]

#3 randomized controlled trial[pt] OR controlled clinical trial[pt] OR clinical trial[pt] OR randomized controlled trials[mh] OR random allocation[mh] OR double-blind method[mh] OR singleblind method[mh] OR random*[tiab] OR random*[tw] OR  ("clinical trial"[tw]) OR drug therapy[sh] OR trial[tiab] OR groups[tiab] OR prospective studies[mh] OR NOT (animal[mh] NOT human[mh]) –

TOTAL #1 AND #2 AND #3

**Cochrane Library**

#1    MeSH descriptor: [hydrochlorothiazide] explode all trees

#2    MeSH descriptor: [chlorothiazide] explode all trees

#3    MeSH descriptor: [bendroflumethiazide] explode all trees

#4    MeSH descriptor: [hydroflumethiazide] explode all trees

#5    MeSH descriptor: [cyclopenthiazide] explode all trees

#6    MeSH descriptor: [trichlormethiazide] explode all trees

#7    MeSH descriptor: [methyclothiazide] explode all trees

#8    MeSH descriptor: [polythiazide] explode all trees

#9    MeSH descriptor: [chlorthalidone] explode all trees

#10  MeSH descriptor: [indapamide] explode all trees

#11  MeSH descriptor: [thiazide diuretics] explode all trees

#12  MeSH descriptor: [mefruside] explode all trees

#13  MeSH descriptor: [xipamide] explode all trees

#14  MeSH descriptor: [clopamide] explode all trees

#15  MeSH descriptor: [triamterene] explode all trees

#16      MeSH descriptor: [spironolactone] explode all trees

#17  MeSH descriptor: [amiloride] explode all trees

#18  MeSH descriptor: [sodium channel blockers] explode all trees

#19  MeSH descriptor: [mineralocorticoide receptor antagonists] explode all trees

#20  dichlothiazide or dihydrochlorothiazide or hctz or butizide or buthiazide or isobutylhydrochlorothiazide or bendrofluazide or trifluoromethylhydrothiazide or cyclothiazide or cyclopenthiazide or cyclomethiazide or chlortalidone or chlorphthalidolone or metolazone or phthalamudine or quinethazone or metolazone or quinethazone or fenquizone or clorexolone or chlorexolone or metindamide or diapamide or bemetizide or benzthiazide or benzothiazide or chlorazanil or thiazide or diuretics, thiazide or benzothiadiazine or sodium chloride symporter inhibitors or sodium chloride cotransporter inhibitor or potassium depleting diuretics or diuretics, potassium depletion or eplerenone or EnaC blocker or inhibitor of the epithelial sodium channel or co-amilozide or coamilozide or mineralocorticoid antagonist or mineralocorticoid receptor antagonist or aldosterone antagonists or potassium sparing diuretic

#21  #1 or #2 or #3 or #4 or #5 or #6 or #7 or #8 or #9 or #10 or #11 or #12 or #13 or #14 or #15 or #16 or #17 or #18 or #19 or #20

#22  MeSH descriptor: [hypertension] explode all trees

#23  “hypertensive patients” or “patients, hypertensive”

#24  #22 or #23

#25  MeSH descriptor: [Randomized Controlled Trial] explode all trees

#26  MeSH descriptor: [Random Allocation] explode all trees

#27  MeSH descriptor: [Randomized Controlled Trials as Topic] explode all trees

#28  double-blind method or controlled clinical trial or clinical trial

#29  #25 or #26 or #27 or #28

#30  #21 and #24 and #29 in Trials

**Embase**

#1 ‘hydrochlorothiazide’/exp OR ‘chlorothiazide’/exp OR ‘bendroflumethiazide’/exp OR ‘hydroflumethiazide’/exp OR ‘cyclopenthiazide’/exp OR ‘trichlormethiazide’/exp OR ‘methyclothiazide’/exp OR ‘polythiazide’/exp OR ‘chlorthalidone’/exp OR ‘indapamide’/exp OR ‘thiazide diuretic agent’/exp OR ‘mefruside’/exp OR ‘xipamide’/exp OR ‘clopamide’/exp OR dichlothiazide OR dihydrochlorothiazide OR hctz OR butizide OR buthiazide OR isobutylhydrochlorothiazide OR bendrofluazide OR trifluoromethylhydrothiazide OR cyclothiazide OR cyclopenthiazide OR cyclomethiazide OR chlortalidone OR chlorphthalidolone OR metolazone OR phthalamudine OR quinethazone OR metolazone OR quinethazone OR fenquizone OR clorexolone OR chlorexolone OR metindamide OR diapamide OR bemetizide OR benzthiazide OR benzothiazide OR chlorazanil OR thiazide OR diuretics, thiazide OR benzothiadiazine OR sodium chloride symporter inhibitors OR sodium chloride cotransporter inhibitor OR potassium depleting diuretics OR diuretics, potassium depletion OR ‘eplerenone’/exp OR ‘triamterene’/exp OR ‘spironolactone’/exp OR ‘amiloride’/exp OR 'sodium channel blocking agent'/exp OR sodium channel blockers OR ‘aldosterone receptor antagonists’/exp OR aldosterone antagonists OR 'potassium sparing diuretic agent'/exp OR potassium sparing diuretic OR EnaC blocker OR ‘inhibitor of the epithelial sodium channel’/exp OR co-amilozide OR coamilozide OR ‘mineralocorticoid antagonist’/exp OR mineralocorticoid receptor antagonist

#2 ‘hypertension’/exp OR ‘hypertensive patient’/exp OR patients, hypertensive OR blood pressure OR ‘systolic blood pressure’/exp OR ‘diastolic blood pressure’/exp

#3 random$ OR doubl$ adj blind$ OR singl$ adj blind$ OR assign$ OR allocat$ OR 'randomized controlled trial'/exp

#1 AND #2 AND #3

**Web of Science**

#1 TS=((hydrochlorothiazide OR chlorothiazide OR bendroflumethiazide OR hydroflumethiazide OR cyclopenthiazide OR trichlormethiazide OR methyclothiazide OR polythiazide OR chlorthalidone OR indapamide OR thiazide diuretic agent OR mefruside OR xipamide OR clopamide OR dichlothiazide OR dihydrochlorothiazide OR hctz OR butizide OR buthiazide OR isobutylhydrochlorothiazide OR bendrofluazide OR trifluoromethylhydrothiazide OR cyclothiazide OR cyclopenthiazide OR cyclomethiazide OR chlortalidone OR chlorphthalidolone OR metolazone OR phthalamudine OR quinethazone OR metolazone OR quinethazone OR fenquizone OR clorexolone OR chlorexolone OR metindamide OR diapamide OR bemetizide OR benzthiazide OR benzothiazide OR chlorazanil OR thiazide OR diuretics, thiazide OR benzothiadiazine OR sodium chloride symporter inhibitors OR sodium chloride cotransporter inhibitor OR potassium depleting diuretics OR diuretics, potassium depletion OR amiloride OR triamterene OR spironolactone OR eplerenone OR sodium channel blockers or aldosterone receptor antagonists or aldosterone antagonists or  potassium sparing diuretic or EnaC blocker OR inhibitor of the epithelial sodium channel OR co-amilozide OR coamilozide OR mineralocorticoid antagonist OR mineralocorticoid receptor antagonist)

#2 TS=((hypertension OR hypertensive patients OR patients, hypertensive OR blood pressure OR systolic blood pressure OR diastolic blood pressure))

#3 TS=((randomized controlled trial OR controlled clinical trial OR clinical trial OR randomized controlled trials OR random OR clinical trial))

**Lilacs**

#1 (tw:(hydrochlorothiazide OR chlorothiazide OR bendroflumethiazide OR hydroflumethiazide OR cyclopenthiazide OR trichlormethiazide OR methyclothiazide OR polythiazide OR chlorthalidone OR indapamide OR thiazide diuretic agent OR mefruside OR xipamide OR clopamide OR dichlothiazide OR dihydrochlorothiazide OR hctz OR butizide OR buthiazide OR isobutylhydrochlorothiazide OR bendrofluazide OR trifluoromethylhydrothiazide OR cyclothiazide OR cyclopenthiazide OR cyclomethiazide OR chlortalidone OR chlorphthalidolone OR metolazone OR phthalamudine OR quinethazone OR metolazone OR quinethazone OR fenquizone OR clorexolone OR chlorexolone OR metindamide OR diapamide OR bemetizide OR benzthiazide OR benzothiazide OR chlorazanil OR thiazide OR diuretics, thiazide OR benzothiadiazine OR sodium chloride symporter inhibitors OR sodium chloride cotransporter inhibitor OR potassium depleting diuretics OR diuretics, potassium depletion OR eplerenone OR amiloride OR triamterene OR spironolactone OR sodium channel blockers OR aldosterone receptor antagonists OR aldosterone antagonists OR  potassium sparing diuretic OR EnaC blocker OR inhibitor of the epithelial sodium channel OR co-amilozide OR coamilozide OR mineralocorticoid antagonist OR mineralocorticoid receptor antagonist) AND (hypertension OR hypertensive patients OR patients, hypertensive OR blood pressure OR systolic blood pressure OR diastolic blood pressure))

#2 (tw:(hypertension OR “hypertensive patients” OR “patients, hypertensive” OR “blood pressure” OR “systolic blood pressure” OR “diastolic blood pressure”))

#3 (db:("LILACS"))

#1 AND #2 AND #3

**Scopus**

#1 KEY(hydrochlorothiazide OR chlorothiazide OR chlorthalidone OR indapamide OR thiazide AND diuretic OR eplerenone OR spironolactone OR triamterene OR amiloride) OR ALL(bendroflumethiazide OR hydroflumethiazide OR cyclopenthiazide OR trichlormethiazide OR methyclothiazide OR polythiazide OR thiazide diuretic agent mefruside OR xipamide OR clopamide OR dichlothiazide OR dihydrochlorothiazide OR hctz OR butizide OR buthiazide OR isobutylhydrochlorothiazide OR bendrofluazide OR trifluoromethylhydrothiazide OR cyclothiazide OR cyclopenthiazide OR cyclomethiazide OR chlortalidone OR chlorphthalidolone OR metolazone OR phthalamudine OR quinethazone OR metolazone OR quinethazone OR fenquizone OR clorexolone OR chlorexolone OR metindamide OR diapamide OR bemetizide OR benzthiazide OR benzothiazide OR chlorazanil OR thiazide OR diuretics, thiazide OR benzothiadiazine OR sodium chloride symporter inhibitors OR sodium chloride cotransporter inhibitor OR potassium depleting diuretics OR diuretics, potassium depletion OR aldosterone antagonists OR EnaC blocker OR co-amilozide OR coamilozide OR mineralocorticoid antagonist OR aldosterone receptor antagonists OR mineralocorticoid AND receptor AND antagonist OR inhibitor AND of AND the AND epithelial AND sodium AND channel OR sodium AND channel AND blockers OR potassium AND sparing AND diuretic)

#2 KEY(hypertension OR hypertensive AND patients)

#3 KEY(randomized AND controlled AND trial OR clinical AND trial) AND NOT review AND NOT (systematic AND review) AND NOT (observational AND study)

**ERIC**

“hypertension”

**Clinical Trials**

Condition or disease: Hypertension

Other terms: Hypertensive patients

Study type: Interventional studies (Clinical trials)

Study results: All studies

Status: “Recruiting”, “active, not recruiting”, “terminated”, “completed” and “unknown status”.

Age: Adult (18-64) and older adult (65+)

Sex: All

Intervention/treatment: Diuretics

Additional criteria: Phase 2, phase 3 and phase 4
